# Bayesian Tractography Using Geometric Shape Priors

**DOI:** 10.3389/fnins.2017.00483

**Published:** 2017-09-07

**Authors:** Xiaoming Dong, Zhengwu Zhang, Anuj Srivastava

**Affiliations:** ^1^Department of Statistics, Florida State University Tallahassee, FL, United States; ^2^The Statistical and Applied Mathematical Sciences Institute (SAMSI), Research Triangle Park Durham, NC, United States; ^3^Department of Statistical Science, Duke University Durham, NC, United States

**Keywords:** tractography, geometric shape analysis, Bayesian estimation, dMRI fiber tracts, active contours

## Abstract

The problem of estimating neuronal fiber tracts connecting different brain regions is important for various types of brain studies, including understanding brain functionality and diagnosing cognitive impairments. The popular techniques for tractography are mostly sequential—tracts are grown sequentially following principal directions of local water diffusion profiles. Despite several advancements on this basic idea, the solutions easily get stuck in local solutions, and can't incorporate global shape information. We present a global approach where fiber tracts between regions of interest are initialized and updated via deformations based on gradients of a posterior energy. This energy has contributions from diffusion data, global shape models, and roughness penalty. The resulting tracts are relatively immune to issues such as tensor noise and fiber crossings, and achieve more interpretable tractography results. We demonstrate this framework using both simulated and real dMRI and HARDI data.

## 1. Introduction

This paper considers an important problem of estimating major white matter fiber tracts in human brain using diffusion magnetic resonance imaging (dMRI) images (Mori et al., 2005). The construction of fiber tracts connecting different brain regions is an important first step toward studying brain connectomics and its implications in assessment of brain functionality, including cognitive abilities and general health. Spurred by experimental development of large databases involving human subjects, with samples across different demographic groups, there is a emerging interest in representing and quantifying brain connectivity patterns. Therefore, efficient and reliable fiber tracking algorithms are urgently needed. However, the problem of estimating fiber tracts using dMRI data is far from being solved (Maier-Hein et al., 2016). The current solutions have many limitations, including inefficiency and susceptibility to noisy, corrupt, and low-quality data. The data mostly comes from pre-processed dMRI images, providing at each voxel a measure of diffusivity of water molecule at that location. The representation of this diffusivity is generally a 3 × 3 symmetric, positive definite matrix (SPDM), also called a *tensor*. In situations where higher resolution data are available, one constructs high angular resolution diffusion imaging (HARDI) data; at each spatial location the orientation diffusion function (ODF, a function on a unit sphere §^2^) is estimated (Descoteaux, 2015). Given these local diffusivity measures, one seeks to form fiber tracts, or their collections in the form of fiber bundles, between regions of interest (ROIs), and to further develops structural networks (Cheng et al., 2012; de Reus and van den Heuvel, 2013; Fornito et al., 2013; Durante and Dunson, 2017). This paper focuses on estimation of fiber tracts, also termed *tractography*, using dMRI and HARDI data. For any two regions (voxels) in a brain coordinate system, the goal is to estimate a collection of curves that follow an optimal pattern of fluid flow connecting these locations, while conforming to anatomical reasonings and interpretations.

Due to the importance of tract-based connectivity in brain connectomic analysis, there have been a number of solutions developed for estimating fiber tracts. They can be loosely grouped into two categories: local and global methods. Local methods construct fiber curves sequentially based on the estimated local diffusion directions. Depending on the mechanism for specifying a local propagation direction, one can further classify the local methods into deterministic methods (Mori et al., 1999; Basser et al., 2000) or probabilistic methods (Hagmann et al., 2003). While the deterministic methods mainly follow the local principal directions to grow fiber curves, the probabilistic methods propose a propagation direction from voxelwise probability distribution, e.g., orientation distribution function (ODF), for growing fibers. The first successful deterministic tractography algorithm was dubbed FACT (fiber assignment by continuous tracking), which has been widely studied in the literature (Mori and van Zijl, 2002). But the limitations of FACT and similar methods are obvious. They include sensitivity to initialization, the susceptibility of principal direction estimation to local noise, and lack of connectivity information between regions of the brain. These limitations drive people to use the probabilistic algorithms. One advantage of the probabilistic methods is that they are based on the full, albeit local, distribution of fiber directions, rather than just the principal direction. They can output a connectivity index measure, e.g., the number of fiber curves, between any two regions of interest, indicating the probability with which the regions are connected to one another. However, this creates problems when the local diffusion directions are not well estimated or are overly smooth. On the other hand, the global methods try to reconstruct fiber curves simultaneously by optimizing the configuration that best matches the given data. Finding the fiber curves that best matches the given data is a hard inverse problem. Current solutions are to translate this inverse problem into a forward problem using a Bayesian approach. For example, Reisert et al. (2011) used a Metropolis Hastings sampler to propose small line segments to fit the given dMRI data and use them to further generate long fiber curves. The global methods provide a better stability with respect to the noise and imaging artifacts. However, there are some issues with the current global methods also. The Bayesian methods typically have high computational cost and require huge memory space, to compute and store a whole ensemble of solutions. Also, in an optimization setting, it is difficult to avoid local solutions since no additional structure is imposed on the optimization.

**Figure 1:**
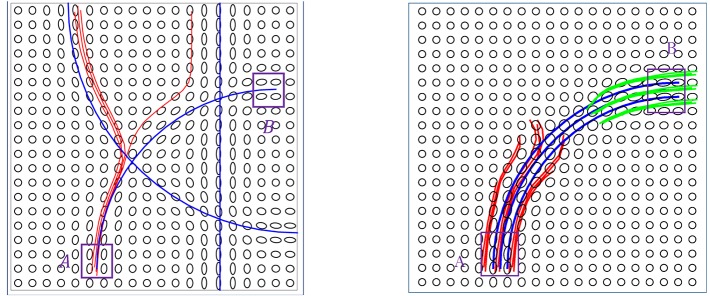
Two examples of the classic streamline method does not work. The blue lines are ground truth fibers. The red and green lines are the tractorgraphy results from the FACT method. Starting from area A, FACT failed to reconstruct the fiber tracts that connect A and B.

We can summarize the limitations of current methods as follows: (a) The local methods are essentially sequential—they start fibers from one end and grow them over time. This one-boundary solution is not natural for tractography, which is actually a two-boundary problem. (b) The local tractography algorithms are highly susceptible to fiber crossing, noise and imaging artifacts. Incorrect recording or noisy observations of tensors can send algorithms in wrong directions and it is difficult to recover from such misdirections. (c) The global tractography algorithms achieve better stability with respect to noise, but they are very computationally expensive. (d) Both local and global methods tend to produce a large proportion of false positive fibers because of the noise and ambiguity at fiber crossings. Figure 1 shows some examples of limitations of a local streamline method, where the blue lines denote ground truth, the red and green lines are tractorgraphy results from the classic FACT method. The left panel shows the challenge of fiber crossing, where the sequential approach fails to reach the target region. The right panel shows the effect of having a patch of noisy data in the middle. The fibers from either regions run into this noisy patch and fail to reach the other end. Additional examples of the challenges faced by streamline methods on the real data, are shown later in the experimental results section. A global approach used for estimating fiber tracts, or curves in general image data, is called *active contours*, where one evolves a curve in order to minimize an energy functional (Pichon et al., 2005; Lankton et al., 2008; Melonakos et al., 2008; Eckstein et al., 2009; Mohan et al., 2009; Zach et al., 2009; Li and Hu, 2013). Other global techniques (Faugeras et al., 2004), including a variation of Kalman Filter (Cheng et al., 2015), have also been applied to this problem.

In this paper, we propose a new approach that is essentially a global method but using additional geometry information for ensuring optimal solutions. The proposed method is fast and easy to implement, and robust to the noise in the data. Most importantly, it can incorporate the prior knowledge from anatomical structure and brain connectomics. Rather than growing fiber tracts sequentially, our idea is to initialize fiber tracts between regions of interest as Euclidean curves and then *deform* them iteratively using gradients of a posterior energy. This approach, termed *Bayesian Active Contours* (Joshi and Srivastava, 2009; Bryner et al., 2013), estimates fiber tracts under an energy function that has contributions from three sources: the given data or the likelihood term, the prior knowledge on the geometric shapes of fibers connecting these ROIs, and a roughness penalty. The algorithm uses the gradient of this posterior energy to iteratively update curves into high probability and highly interpretable fiber tracts. The prior on the geometric shapes relies on developing statistical shape models of fiber curves between ROIs, using atlas data, and evaluating expressions for gradient of resulting shape model energy with respect to the shape variable. We use advances in elastic shape analysis of Euclidean curves to develop efficient statistical models for fiber bundles using training (or atlas) data. The training data can be generated using existing local or global tractography algorithms, or can use manual inputs. These models form prior information for future tractography and, in conjunction with diffusion data likelihood, they provide tract estimation results.

In contrast to the probabilistic tractography method (Behrens et al., 2003, 2007), the proposed Bayesian method is a global one. We start with an initial fiber connecting two pre-specified regions and update it under an energy function. The final fiber can best explain the diffusion data under the constraints of prior shape distribution and desired smoothness. Previously, there are some Bayesian tractography methods proposed in the literature (Friman et al., 2006; Cook et al., 2008; Yap et al., 2011). These methods are different from the proposed one: in our method, we assign a prior on the fiber shape space, while in (Friman et al., 2006; Cook et al., 2008; Yap et al., 2011), the prior is imposed on local fiber orientation distribution. Probably, the most similar work to ours is (Christiaens et al., 2014), where an atlas-guided global tractography is introduced with a prior on the local tract distribution. However, our work is different in two aspects: Firstly, we have a different energy function. We introduce a novel data term and a smoothness term separately to measure alignment between fibers and diffusion data, and the smoothness of fiber tracts. Secondly, we have a different prior. We incorporate the prior information of fiber shape from the atlas space while (Christiaens et al., 2014) obtains the prior information of local tract distribution from the atlas space.

The rest of this paper is organized as follows. We describe the three components of the posterior energy—data likelihood, shape prior and roughness penalty—and their gradients in Section 2. The resulting tractography algorithm is laid out in Section 3, and experimental results using both simulated and real data, the extension to HARDI data are presented in Section 4. We close the paper with a short discussion in Section 5.

## 2. Mathematical framework for Bayesian tractography

Although the framework can be easily generalized to 3D data, we will restrict to 2D data in this paper for simplicity. The theory is general enough to be applicable to 3D data directly.

First, we develop a mathematical framework for estimation of fiber tracts using tensor data and prior shape models. Let P be the set of 2 × 2 symmetric, positive definite matrices (or tensors). For the domain, *D* = [0, 1]^2^, let *M* : *D* → P denote a continuous vector field of tensors defined on this domain. Let β:[0, 1] → *D* be an absolutely continuous curve contained in this domain, and let B be the set of all such curves. Our goal is to find a β with certain boundary constraints that optimizes a chosen objective function that comes from a Bayesian formulation. Thus, we pose the problem of tractography as a MAP estimation. In this formulation we seek parameterized curve $\hat{\beta }$ that minimizes an energy functional according to: $\hat{\beta }={\text{argmin}}_{\beta \in B}\;E_{total}\left(\beta \right)$, where

(1) $$\begin{array}{l} E_{total}(\beta )=\lambda_{1}E_{data}(\beta )+\lambda_{2}E_{prior}(\beta )+\lambda_{3}E_{smooth}(\beta ). \end{array}$$

This total energy functional has contributions from three different criteria that are weighted by the coefficients λ_1_, λ_2_, λ_3_ > 0. The data energy *E*_*data*_ is defined solely from the diffusion data in the image, *E*_*prior*_ is the prior shape energy defined from a statistical model on shapes of the fiber β, and the smoothing energy *E*_*smooth*_ is a penalty that ensures a certain amount of smoothness in the estimated fiber. In order to minimize E_total_ we use a gradient descent procedure that updates the curve according to β ↦ β − δ∇_β_*E*, where

(2) $$\begin{array}{l} \nabla_{\beta }E=\lambda_{1}\nabla E_{data}(\beta )+\lambda_{2}\nabla E_{prior}(\beta )+\lambda_{3}\nabla E_{smooth}(\beta ). \end{array}$$

That is, we search for a local minimization of Equation (1) via gradient descent. The weights λ_*i*_ will certainly affect curve evolution, i.e., a large penalty on the smoothness term favors shorter fibers and so on. Through trial and error, one can adjust the λ's depending on the data and problem context. In the next three sections, we summarize the formulation of each of the three energy terms.

### 2.1. Data-likelihood term

**Figure 2:**
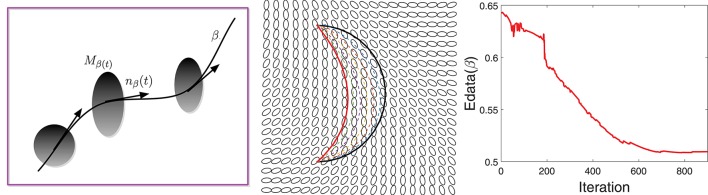
**(Left)** A schematic showing a curve β passing through a tensor field *M*. **(Middle)** An example of gradient-based optimization under *E*_*data*_, where black is the initial curve and red is the final curve. **(Right)** The evolution of *E*_*data*_ during this optimization.

The data term is designed to quantify the agreement of the fiber directions with the diffusion tensor at that location. Let *M* be a given tensor field and β be a curve lying in the domain *D*, as shown in the left panel of Figure 2. The data energy term is then given by:

(3) $$\begin{array}{l} E_{data}[\beta ]={\int_{0}^{1}n_{\beta }\left(t\right)}^{T}M_{\beta (t)}^{-1}n_{\beta }(t)\;\;dt,\text{where }n_{\beta }(t)=\frac{\dot{\beta }(t)}{\left|\dot{\beta }(t)\right|}. \end{array}$$

Here *n*_β_(*t*) denotes the unit vector tangent to β at β(*t*) and *M*_β(*t*)_ is the tensor at location β(*t*) ∈ *D*. The integrand is lower at the locations where the fiber tract is aligned with the tensor field and vice-versa.

We motivate the choice of this expression by focusing on some Riemannian frameworks used in tractography:

- **Maximal Curves Matching the Given Tensor Field**: One generally wants to find curves such that their velocity vectors maximally match the given diffusion tensors. Therefore, one may consider maximizing the term:
  $$\begin{array}{l} L_{M}[\beta ]=\int_{0}^{1}\sqrt{\left(\dot{\beta }{\left(t\right)}^{T}M_{\beta (t)}\dot{\beta }(t)\right)}\;\;dt=\int_{0}^{1}|\dot{\beta }(t){|}_{M_{\beta (t)}}\;\;dt. \end{array}$$
  This quantity is nothing but the length of a curve β in *D* under a Riemannian metric defined by the tensor field *M*. The maximizers of *L*_*M*_ are the longest paths between given points in *D*. However, the problem with this is that there is no upper bound on the length of the curve, and one can place arbitrarily long curves in *D* irrespective of *M*.
- **Geodesics Under Inverse Tensor Field**: A better idea is to use the inverse of the given tensor field at each point and then construct geodesic paths under that Riemannian metric (O'Donnell et al., 2002; Duncan et al., 2004; Melonakos, 2009), according to:
  $$\begin{array}{l} \beta^{*}={\text{argmin}}_{\beta }(\int_{0}^{1}\sqrt{\left(\dot{\beta }{\left(t\right)}^{T}M_{\beta (t)}^{-1}\dot{\beta }(t)\right)}\;dt\\ \;=\int_{0}^{1}|\dot{\beta }(t){|}_{M_{\beta (t)}^{-1}}\;dt)\;. \end{array}$$
  One can solve the optimization problem by minimizing an energy, without the square-root in the integrand, as follows:
  $$\begin{array}{l} \beta^{*}={\text{argmin}}_{\beta }\left(\int_{0}^{1}\dot{\beta }{\left(t\right)}^{T}M_{\beta (t)}^{-1}\dot{\beta }(t)dt\right). \end{array}$$
  This way one gets shortest curves such that their velocities agree with the dominant directions of the original tensor field. This formulation also agrees with a probabilistic approach where one uses the tensor field to define a Gaussian distribution at each point (Lenglet et al., 2004), and seeks maximum likelihood estimates. Although this method favors fiber directions similar to the dominant eigen vectors of the given tensor field, it additionally penalizes the lengths of the such fibers. Similar to the previous bullet, it may be possible to find shorter paths that do not agree with the tensor field. Some other papers (Fuster et al., 2014). Hao et al. (2014) have expressed this exact issue in different terms, citing the inability of this method to handle high curvature regions. They proposed a solution based on modifying the Riemannian metric by a curvature-based scalar field and then constructing geodesic paths (Hao et al., 2014). The real issue in these ideas is that there is no independent way to control the lengths of estimated fibers.
- **Scale-Invariant Optimal Paths**: We take a different approach where the length of the fibers is separated from the agreement of fiber directions with the given tensor directions. We weight these two quantities differently and are able to better control the length of the fibers. For the domain *D*, and a given tensor field *M* : *D* → P, we define an energy term given by
  (4) $$\begin{array}{l} E_{data}[\beta ]={\int_{0}^{1}n_{\beta }\left(t\right)}^{T}M_{\beta (t)}^{-1}n_{\beta }(t)\;\;dt, \end{array}$$
  where $n_{\beta }\left(t\right)=\dot{\beta }\left(t\right)/|\dot{\beta }\left(t\right)|$. Note that if we scale the speed of traversal along β by a constant, the energy function remains unchanged. In other words, the integrand only depends on the agreement of the direction *n*_β_(*t*) with the dominant eigen vectors of *M*_β(*t*)_, and not on the speed of traversal at β(*t*). However, this energy function is not invariant to a re-parameterization of β. Let γ : [0, 1] → [0, 1] be a positive diffeomorphism, the β ◦ γ represents a re-parameterization of β. It can be seen that, in general, *E*_*data*_[β] ≠ *E*_*data*_[β ◦ γ]. If that invariance is desired, one can achieve it by changing the measure of integration from *dt* to $|\dot{\beta }\left(t\right)|dt$ in Equation (4).

The next step is to derive the gradient of *E*_*data*_ with respect to β for use in gradient-based optimization. To specify the gradient of *E*_*data*_, we need some additional notation. Note that for any location *x* = (*x*_1_, *x*_2_) ∈ *D*, the gradient of *M* : *D* → P has two components, ∇_*x*_1__*M*_*x*_, ∇_*x*_2__*M*_*x*_ ∈ *T*_*M*_*x*__(P). Thus, the gradient vector ∇_*x*_*M*_*x*_ is a higher-order tensor of the size 2 × 2 × 2. For any such tensor *A* ∈ ℝ^2 × 2 × 2^ and a vector *x* ∈ ℝ^2^, we will use the notation: 〈〈*A, x*〉〉 to imply *x*_1_*A*(:, :, 1)+*x*_2_*A*(:, :, 2) ∈ *T*_*M*(*x*)_(P). Therefore, 〈(〈〈*Ax*〉〉)*x*〉 denotes a 2-vector given by $x_{1}A\left(:,:,1\right)x+x_{2}A\left(:,:,2\right)x\in {\mathbb{R}}^{2}$. With this notation, we can express the gradient of *E*_*data*_ as follows.

**LEMMA 1**. *The gradient of E_data_ with respect to* β, *under the* 𝕃^2^ *norm, is given by:*

(5) $$\begin{array}{l} -2\{\frac{1}{\left|\dot{\beta }(t)\right|}\left(M_{\beta (t)}^{-1}{\dot{n}}_{\beta }(t)+\langle \langle \nabla_{x}M_{\beta (t)}^{-1},\dot{\beta }(t)\rangle \rangle n_{\beta }(t)\right)\\ \;-\frac{{\dot{\beta }}^{T}(t)\ddot{\beta }(t)}{|\dot{\beta }(t){|}^{3}}M_{\beta (t)}^{-1}n_{\beta }(t)-\frac{1}{\left|\dot{\beta }(t)\right|}({\dot{n}}_{\beta }(t)n_{\beta }^{T}(t)M_{\beta (t)}^{-1}n_{\beta }(t)\\ \;+n_{\beta }(t)n_{\beta }^{T}(t)\langle \langle \nabla_{x}M_{\beta (t),}^{-1}\dot{\beta }(t)\rangle \rangle n_{\beta }(t)+2n_{\beta }(t)n_{\beta }^{T}(t)M_{\beta (t)}^{-1}{\dot{n}}_{\beta }(t))\\ \;+\frac{{\dot{\beta }}^{T}(t)\ddot{\beta }(t)}{|\dot{\beta }(t){|}^{3}}n_{\beta }(t)n_{\beta }^{T}(t)M_{\beta (t)}^{-1}n_{\beta }(t)\}+\langle \langle tran(\nabla_{x}M_{\beta (t)}^{-1}),n_{\beta }(t)\rangle \rangle n_{\beta }(t). \end{array}$$

where $tran\left(\nabla_{x}M_{\beta \left(t\right)}^{-1}\right)$ is transpose of $\nabla_{x}M_{\beta \left(t\right)}^{-1}$.

A derivation of this expression is presented in the **Appendix**. Having an analytical expression for ∇_β_*E*_*data*_ makes the optimization problem more efficient, as compared to purely numerical solutions.

Figure 2 shows an example of the gradient-based minimization of E_data_ in the middle panel. It shows a tensor field *M* and an initial curve β (in black). We update β iteratively using −∇_β_*E*_*data*_ and the result is drawn as a red curve. The corresponding evolution of *E*_*data*_ is plotted in the right panel.

### 2.2. Smoothness or fiber length term

**Figure 3:**
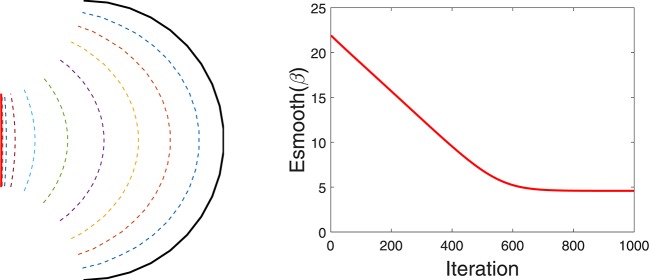
Evolution of a curve using negative gradient of *E*_*smooth*_. **(Left)** The initial curve in black, intermediate curves as dotted lines, and the final curve in red. **(Right)** The evolution of *E*_*smooth*_.

For regulating smoothness of the estimated curve, we follow a common approach from geometric active contours that is motivated in part by Euclidean heat flow. Define the smoothing energy function as $E_{smooth}\left(\beta \right)=\int_{0}^{1}|\dot{\beta }\left(t\right)|dt$, which is equal to the length of the curve and is naturally invariant to any re-parameterization. It is shown in Kichenassamy et al. (1995) that the gradient of *E*_*smooth*_ is given by the Euclidean heat flow equation ∇*E*_*smooth*_(β) = κ_β_**n**_β_, where κ_β_ is the curvature at each point of β and **n**_β_ is the unit normal field along the curve. It is well known that this particular penalty on a curve's length leads to simultaneous smoothing and shrinking of a curve. If we rescale the curve to keep the original length, the main effect is that of smoothing. An example of this idea is illustrated in Figure 3 that shows a curve evolving according to −∇*E*_*smooth*_. The left panel shows the initial curve (in black), and its updates using the negative gradient of *E*_*smooth*_. The corresponding decrease in *E*_*smooth*_ is plotted on the right.

### 2.3. Atlas-based shape prior

The next term to consider is *E*_*prior*_ that forces the shapes of estimated fiber tracts to be similar to certain desired shapes. This term encodes the prior shape information about fibers connecting two ROIs, and is based on a statistical model that is learnt from the training or atlas data (generated by current local or global methods). In a brain connectome study framework, the brain is generally pre-segmented into small anatomical regions using software such as Freesurfer and ANTs (Avants et al., 2011), and fibers connecting two ROIs are extracted. However, due to differences in sizes, orientations, and coordinate systems, these fibers connecting the same ROIs across subjects can not be directly used as prior for future fiber tractography. Removing these nuisance variable requires a formal definition of shape and shape space, and then one needs to develop a statistical model on this mathematical representation. Here we use elastic shape analysis developed in Srivastava and Klassen (2016) to represent and model fiber shapes. Specifically, we define S, the shape space of all curves in *D* and impose a truncated wrapped normal distribution on this space to reach a statistical shape model. The parameters of this model are estimated *a priori* from the training or atlas data. We present a brief summary of the elastic shape analysis here and refer the reader to the textbook (Srivastava and Klassen, 2016) for more details. For a curve β : [0, 1] → *D*, define $q\left(t\right)=\dot{\beta }\left(t\right)/\sqrt{\left|\dot{\beta }\left(t\right)\right|}$ be the *square-root velocity function* (SRVF) of β. This SRVF representation has an important property that a re-parameterization invariant Riemannian metric on the space of curves becomes the simple 𝕃^2^ metric under transformation. As a corollary, for any *q*_1_, *q*_2_ ∈ 𝕃^2^, we have ‖(*q*_1_, γ) − (*q*_2_, γ)‖ = ‖*q*_1_ − *q*_2_‖, for any γ ∈ Γ, where Γ is the set of all orientation preserving diffeomorphisms of [0, 1]. Here (*q*, γ) stands for $\left(q\circ \gamma \right)\sqrt{\overset{\cdot }{\gamma }}$, representing the SRVF of the re-parameterized curve β ∘ γ. If we rotate β by *O* ∈ *SO*(2), we get O^*^β, and the corresponding SRVF is given by O^*^q.

Let β be a rescaled fiber curve such that it has unit length and let *q* be its SRVF. We define an orbit in the SRVF space as $\left[q\right]=\left\{O\left(q\circ \gamma \right)\sqrt{\overset{\cdot }{\gamma }}|O\in SO\left(2\right),\gamma \in \Gamma \right\}$, which denotes an equivalence class representing a shape. Let S be the set of all such equivalence classes; S is called the shape space. The term *E*_*prior*_ in the active contour model is a function of β, but our statistical models are built on S such that *E*_*prior*_ can effectively encode the shape information and be invariant to the different sizes and coordinate systems of different brains. However, S is a nonlinear manifold space. To build a statistical model on S, we need some elementary tools such as efficient methods to calculate the mean and covariance matrix for a given set of data. Here we employ Karcher mean to calculate the mean shape of given fiber curves and the covariance matrix is calculated on the tangent space of S at the estimated Karcher mean denoted by *T*_[μ]_(S). The reader can refer to Srivastava et al. (2011) for the explicit procedures to calculate the Karcher mean and the covariance matrix.

Given a set of prior training shapes {[*q*_*i*_], *i* = 1, …*n*} in S, let us assume that we have computed their Karcher mean [μ] and covariance *K*. We define the prior shape model using a *truncated wrapped-normal density*, which is estimated from the data as follows. First, obtain the singular value decomposition of *K* as [*U, S, V*] = svd(*K*), and let *U*_*m*_ be the *m*-dimensional principal subspace of $T_{\left[\mu \right]}\left(S\right)$ spanned by the first *m* columns of *U*. The shape prior distribution is defined as a wrapping of the truncated normal distribution mapped from *U*_*m*_ to S using the exponential map. The truncated normal density on *U*_*m*_ is:

(6) $$\begin{array}{l} v\sim \frac{1}{Z}e^{-\frac{1}{2}\left(v_{\|}^{T}S_{m}^{-1}v_{\|}+\|v_{⊥}{\|}^{2}/\delta^{2}\right)}1_{\|v\|<\pi }, \end{array}$$

where $v=\exp_{\left[\mu \right]}^{-1}\left(\left[q\right]\right)$, $v_{\left|\right|}=U_{m}^{T}v$ is the projection of *v* into *U*_*m*_, *v*_⊥_ = *v* − *U*_*m*_*v*_‖_, *S*_*m*_ is the diagonal matrix containing the first *m* singular values, and *Z* is the normalizing constant. The scalar value δ is chosen to be less than the smallest singular value in *S*_*m*_. Suppose now that we have a test shape [*q*] that represents a fiber tract during optimization process, and $v=\exp_{\left[\mu \right]}^{-1}\left(\left[q\right]\right)$ be the shooting vector from the mean [μ] to [*q*]. Now define *E*_*prior*_(*q*) to be the negative of the exponent in the shape prior given by Equation (6). That is, define $E_{prior}\left(q\right)=\frac{1}{2}v^{T}\left(U_{m}S_{m}^{-1}U_{m}^{T}\right)v+\frac{1}{2\delta^{2}}||v-U_{m}U_{m}^{T}v|{|}^{2}$. Minimizing this functional is, therefore, equivalent to maximizing the likelihood of *q* under the chosen shape model. The gradient of *E*_*prior*_ with respect to *v* is equal to *w* = *Av*, where *A* is the matrix $A=U_{m}S_{m}^{-1}U_{m}^{T}+\left(I-U_{m}U_{m}^{T}\right)/\delta^{2}$. Notice that *w* is defined on the tangent space at μ rather than at *q*, so the final step is to parallel translate *w* from μ to *q*. Denote this parallel translation as $\bar{w}=\nabla_{q}E_{prior}\left(q\right)$. An evolution of *q* along the negative gradient direction will result in an energy minimization precisely at the mean μ. The translated shooting vector $\bar{w}$ now represent the gradient of *E*_*prior*_ with respect to *q*. As the last step, this gradient is converted to ∇_β_*E*_*prior*_(β) using a numerical approximation.

**Figure 4:**
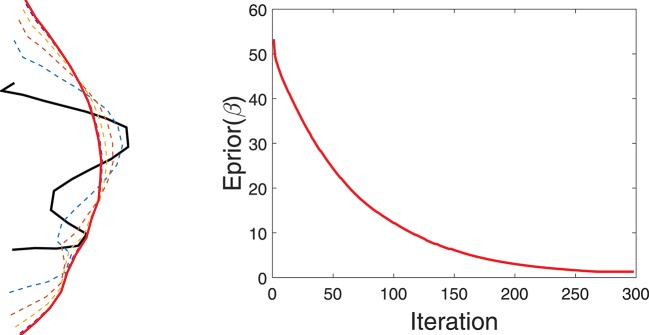
Evolution of a curve using negative gradient of *E*_*prior*_. **(Left)** The initial curve in black, intermediate curves as dotted lines, and the final curve in red. **(Right)** The evolution of *E*_*prior*_.

Figure 4 shows a simple example of evolving a curve according to *E*_*prior*_. The left panel shows the initial curve, and its updates using the negative gradient of *E*_*prior*_. The corresponding decrease in *E*_*prior*_ is plotted on the right.

## 3. Bayesian tractography algorithm

When we put together the three components of the energy, the shape of β is controlled by gradients of *E*_*data*_, *E*_*prior*_ and *E*_*smooth*_, the smoothness is controlled by *E*_*prior*_ and *E*_*smooth*_, and the nuisance variables (placement, scale, and rotation) are controlled only by *E*_*data*_. Now we summarize the overall algorithm for Bayesian tractography using the tensor field (Algorithm 1).

**Algorithm 1:**
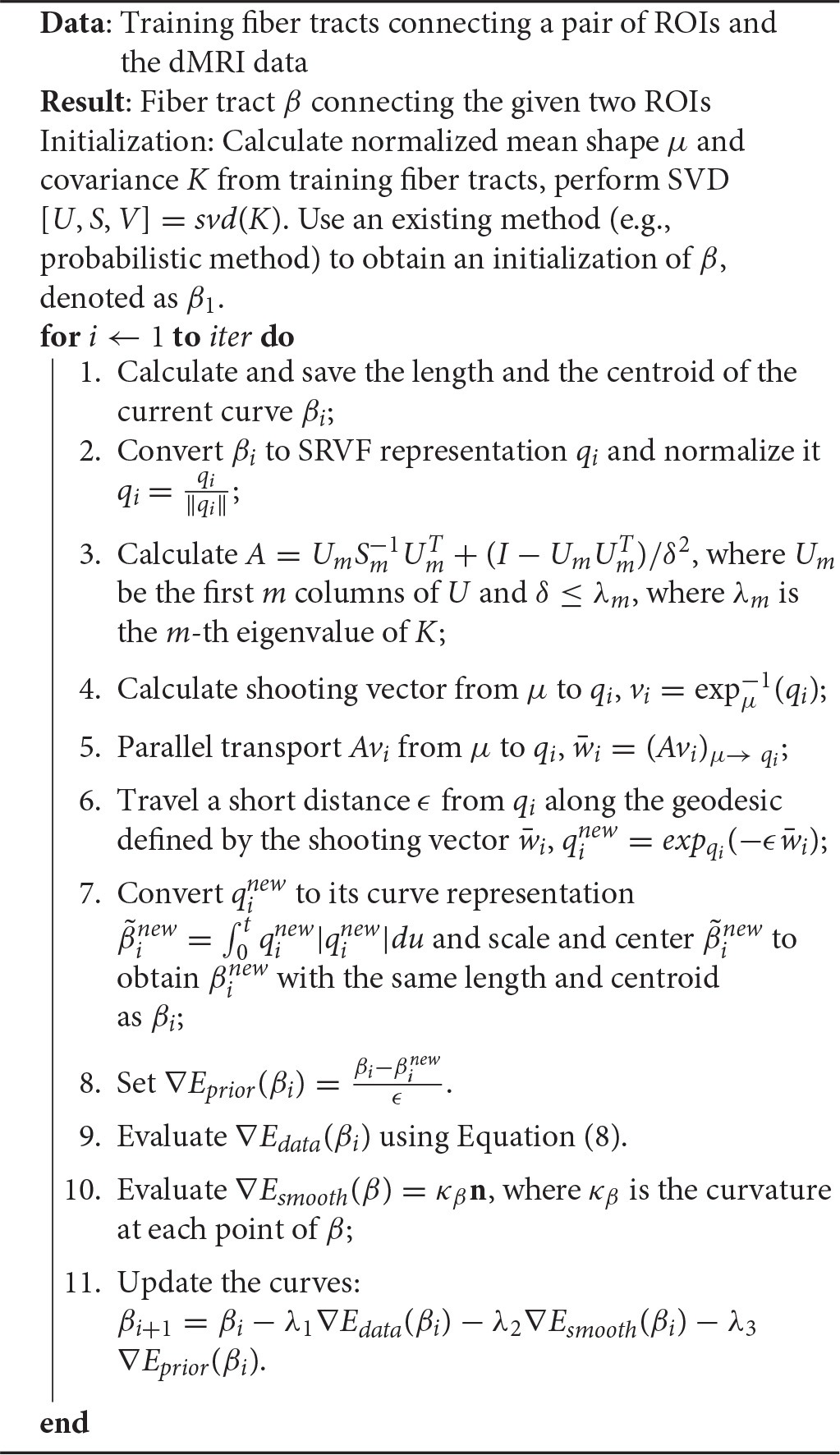
Bayesian Tractography Using Geometric Shape Priors

The advantage of the proposed framework is that it uses a global optimization to overcome issues such as fiber crossing and spatial noise. The final tracking result depends not only on the diffusion data, but also on prior shape information. The inclusion of shape prior distinguishes our method from other energy minimization based fiber-tracking algorithms, and is essential for the optimization procedure to come out of local solutions and reach a global solution. Most importantly, in our framework, the brain is parcellated into small regions, and the shapes of fibers connecting any pair of regions are found to be consistent. The proposed truncated wrapped-normal distribution can effectively capture the variation of shapes for each connection in the training data. In addition, since we reconstruct the whole fiber simultaneously by minimizing an energy function, the issue of fiber crossing has almost no detrimental effect of our fiber tracking algorithm.

As stated earlier, this Bayesian approach requires either a the training data or an atlas of fiber tracts between regions of interest, to estimate shape model and develop *E*_*prior*_. We can construct such data using existing tractography algorithms with maybe human inspection for quality control. However, since such a construction is needed only once, it can be performed offline.

## 4. Experimental results

In this section we present some results using both simulated and real data to illustrate the performance of the proposed method.

### 4.1. Simulated 2-D tensor data

We first study our proposed tracking algorithm in the simulated settings. Let domain *D* = [0, 1]^2^ for all our simulation examples. The tensor field on *D*, denoted by *M*:*D*→P, is generated using certain fibers that play the role of ground truth. We discretize the domain *D* into a 20 × 20 grid, and the tensor within each grid is decided by the tangent directions of the line segments within this grid. In addition, a 2D Gaussian smoothing is applied to smooth the tensor field before applying our algorithm.

**Figure 5:**
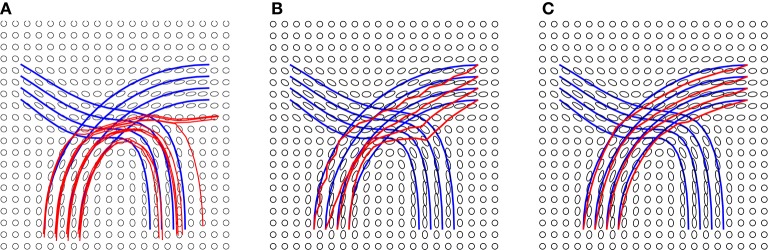
Tractography results on a simulated tensor field and distances from ground truth: **(A)** Streamline tractography from either region, *d* = 1.5*e* − 2, **(B)** our method without a shape prior, *d* = 2.2*e* − 3, and **(C)** our method with a shape prior, *d* = 3*e* − 4. The details of the prior are presented in Figure 6.

In the experiment presented in Figure 5, we use the blue lines as ground truth fiber tracts and generate a tensor field as shown in these panels. Then, using this tensor data, we estimate the fiber tracts using our and other methods, and the results are shown in red lines. On the left side we show results from standard streamline tractography, using starting points on one end. Due to a crossing of fibers in the middle, these tracts get diverted and sent to wrong directions. In the middle panel, we show results from our method but without using the shape prior term. This time the end points of the tracts are correct (by initialization) but some of the fibers don't quite reach the desired shape. Finally, we optimize fiber tracts using the full energy functional, including the shape prior, and display these results in the right panel. By including all the three terms, we overcomed issues caused by fiber crossing and local noise, and reached correct global structures. To better evaluate the tractography results, we calculate the distance between reconstructed fibers and ground truth using the *L*_2_ norm. We first calculate the distance of each fiber from the ground truth and then use the mean of all distances to quantify the difference between reconstructed fiber bundle and ground truth fiber bundle. The distances for each method are given in Figure 5.

**Figure 6:**
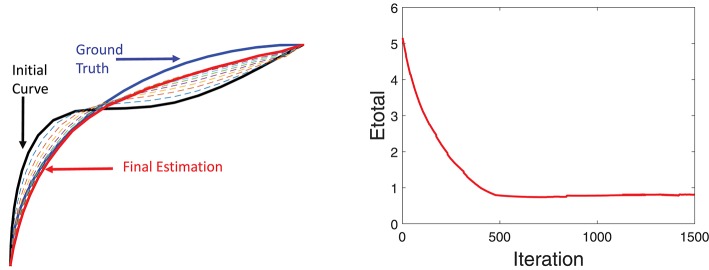
Detailed tractography results in the simulation example. Here we only focus on reconstruction of one of the curves. The black line is initialization, the red line is our result and the blue line is the ground truth.The right panel shows the evolution of the energy function.

Additional details of this simulation experiment are presented in Figure 6, which shows evolution of a single fiber under *E*_*total*_. The left panel shows the initial curve (black), the final curve (red), and the ground truth curve (blue). The right panel shows the evolution *E*_*total*_ during this iteration. In this experiment, we used the weights λ_1_ = 0.8, λ_2_ = 0.1, and λ_3_ = 0.1.

### 4.2. Experiments using real data

**Figure 7:**
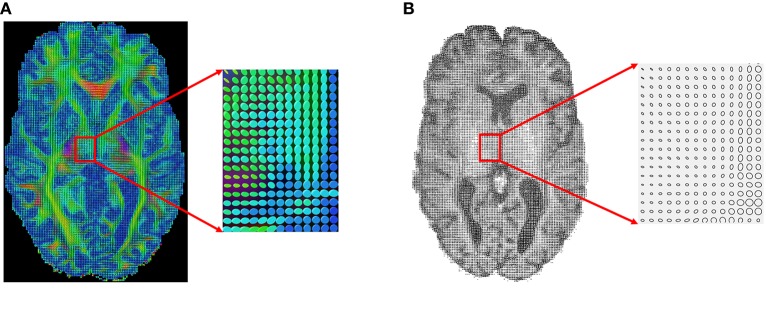
An example of a sagittal slice of diffusion tensor data. **(A)** Original data. **(B)** Projected 2D data.

Next, we apply our method to some real datasets—dMRI images downloaded from the Human Connectome Project (HCP) (Van Essen et al., 2012). HCP contains about 900 subjects with diffusion MRI, but here we have used only 30 subjects for our experiments. The dMRI images in HCP has an isotropic resolution of 1.25 mm. To estimate a diffusion tensor at each voxel, we use the open source software Dipy (Garyfallidis et al., 2014). Figure 7A shows one slice of the 3 × 3 diffusion tensors estimated from a randomly selected dMRI image in HCP; a zoom-in of a small part of the image is shown on its right. Since in this paper we restrict to a 2D domain to illustrate our idea, we convert 3 × 3 diffusion tensors in the original data to 2 × 2 tensors by removing the diffusion directions perpendicular to the 2D slice plane. Figure 7B shows an example of this projection and shows the 2D tensors in form of their level sets or ellipses at each pixel location.

**Figure 8:**
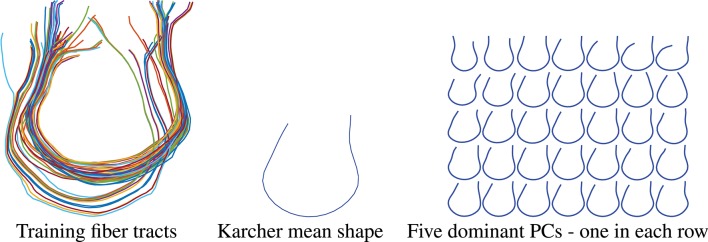
Thirty training samples of fiber tracts, their Karcher mean and principal directions of shape variation. The rightmost panel from top to bottom represents the first 5 principal directions of variation in the training data.

In the results presented here, we focus on estimating a set of fiber curves connecting the left and right superior frontal gyri. In order to generate a prior shape model, we use tracts extracted for 30 subjects between these regions as the training dataset. These tracts were manually identified with the help of Freesurfer Destrieux Atlas (Destrieux et al., 2010) and the fiber curves built using the FACT method. These fibers are displayed on the left side of Figure 8. The Karcher mean μ of these fibers in the shape space S is shown in the middle panel and the five dominant principal components of the Karcher covariance are displayed in the right panel. These dominant directions are computed by projecting the given shapes [*q*_*i*_] in the tangent space *T*_[μ]_(S) using the inverse exponential map, i.e., $v_{i}=\exp_{\left[\mu \right]}^{-1}\left(\left[q_{i}\right]\right)$, and the computing principal components of the set {*v*_*i*_} in the vector space *T*_[μ]_(S). These principal directions, which as straight lines in *T*_[μ]_(S) passing through [μ] in the middle, are then wrapped back on S using the exponential maps. Each row of the right panel in Figure 8 shows plots one such direction, going from the largest variability to smallest from top to bottom.

**Figure 9:**
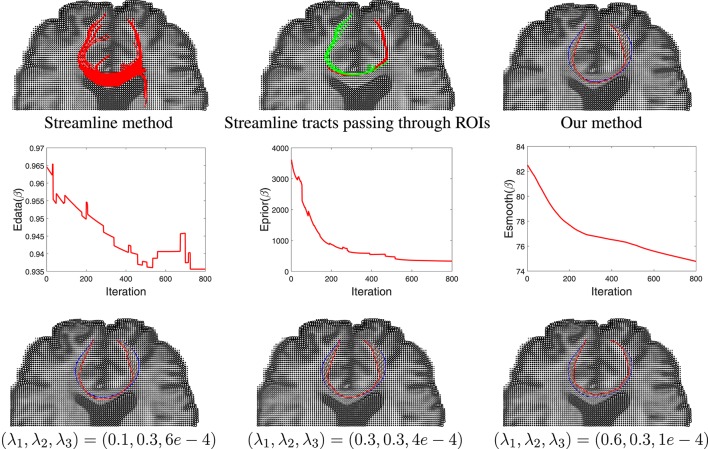
Results comparison between streamline method and our method. In the **top** row, the **left** panel shows the results using a streamline method, the **middle** panel shows some selected curves from that set that reach the two ROIs (different colors represent curves passing different regions), and the **right** panel shows tractography result using our Bayesian method. Here the blue line shows the initialization and red line is final result. The **middle** row shows the evolution of the three energy components in this estimation. The **bottom** row shows our tractography results under different weights of the energy components.

Having developed a prior model for fiber shapes from the training data, we then apply our Bayesian method to the tensor data, especially focusing on the areas where the streamline method fails, and the results are presented in Figure 9. We first show the results of the streamline method, using seeds from either ROI, in the first two panels. While the left panel in the top row gives an appearance that we have some fibers connecting the two ROIs, a closer look shows that this is actually not the case. In the middle panel we color the curves differently depending on which ROI is the seed located in. One can see that the set of curves—red and green—do not not reach the other ROI. They start from the ROI containing the seeds and diverge in the middle. This is in contradiction to the anatomical knowledge that the two regions are indeed connected through white matter fiber tracts. Using the proposed Bayesian technique, we obtained result shown in the rightmost panel of the top row. This picture shows an arbitrarily initialized curve drawn in blue, and the final estimated curve drawn in red color. The corresponding evolutions of the three energy terms—*E*_*data*_, *E*_*prior*_, and *E*_*smooth*_—are shown in the middle row of this figure. Each one of these terms show a substantial decrease in their values during the iteration process.

In order to study the impact of the weights λ_1_, λ_2_, and λ_3_ on the final result, we generated estimates for a few different combinations of these weights. The results are shown in the last row of this figure. In case where the weight for shape prior is high, the final result is close to the prior mean. In contrast, when the weight for the data term is high, there is a better agreement between the curve and the tensor field.

**Figure 10:**
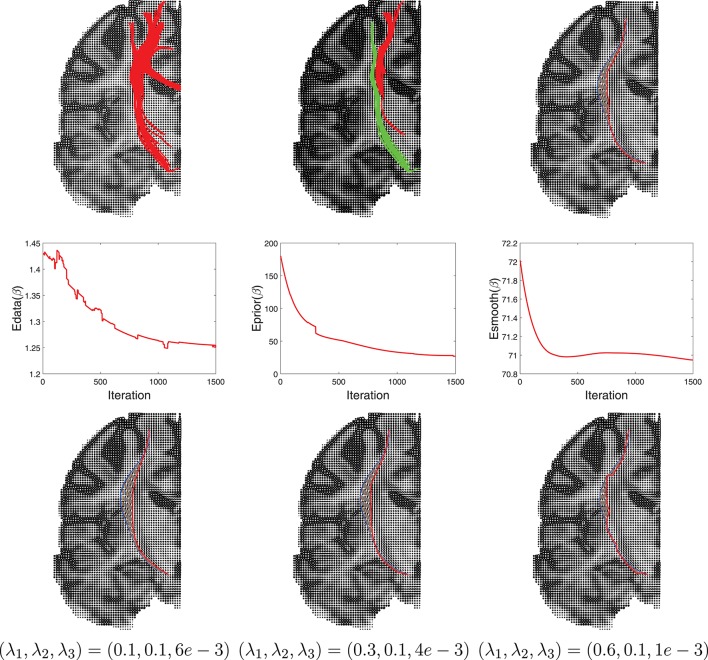
Another example similar to Figure 9.

Another example of this Bayesian estimation is presented in Figure 10 with similar settings. In this case the ROIs used are right hippocampus and right percentral.

### 4.3. Extension to tractography using HARDI data

The proposed framework can be extended to HARDI data, where an ODF is used to better represent the underlying diffusion profile. The data term is now defined as:

(7) $$\begin{array}{l} E_{data}[\beta ]=\int_{0}^{1}-f_{\beta (t)}(n_{\beta }(t))\;\;dt,\text{ where }n_{\beta }(t)=\frac{\dot{\beta }(t)}{\left|\dot{\beta }(t)\right|}. \end{array}$$

Here *n*_β_(*t*) denotes the unit vector tangent to β at β(*t*) and *f*_*p*_ is the ODF at *p* ∈ *D*. The integrand is low at a location where the fiber tract is aligned with the ODF field and vice-versa. The next step is to derive the gradient of *E*_*data*_ with respect to β for use in gradient-based optimization. we can express the gradient of *E*_*data*_ as follows.

**LEMMA 2**. *The gradient of E_data_ with respect to* β, *under the* 𝕃^2^ *norm, is given by:*

(8) $$\begin{array}{l} -\frac{{\dot{\beta }}^{T}(t)\ddot{\beta }(t)}{|\dot{\beta }(t){|}^{3}}\left(I-n_{\beta }(t)n_{\beta }^{T}(t)\right)\nabla_{n_{\beta }}^{T}f_{\beta (t)}(n_{\beta }(t))-\frac{2}{\left|\dot{\beta }(t)\right|}{\dot{n}}_{\beta }(t)n_{\beta }^{T}(t)\nabla_{n_{\beta }}^{T}f_{\beta (t)}(n_{\beta }(t))\\ \;+\frac{1}{\left|\dot{\beta }(t)\right|}\left(I-n_{\beta }(t)n_{\beta }^{T}(t)\right)\nabla_{n_{\beta }}^{2}f_{\beta (t)}(n_{\beta }(t)){\dot{n}}_{\beta }(t). \end{array}$$

**Figure 11:**
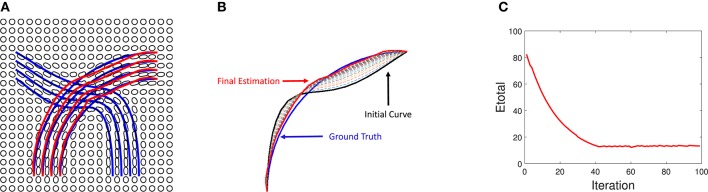
Tractography results on simulated ODF data. **(A)** Red lines are reconstructed fibers using our method and blue lines are the ground truth used to generate the ODF field. **(B)** Evaluation of one curve under our method. **(C)** Evolution of energy term *E*_*total*_.

A derivation of this expression is presented in the **Appendix**. We also show an experiment result on an ODF data in Figure 11. We use the blue lines as ground truth fiber tracts and generate ODF data as shown in Figure 11A. Under this ODF field, we estimate the fiber tracts using our method. The final reconstructed tracts are shown in red lines. In the middle panel, we show an evolution of a single fiber under *E_total_*. In the right panel, we show the evolution *E_total_* of each iteration.

## 5. Conclusion and discussion

This paper introduces a Bayesian approach for estimating fiber tracts, between given pairs of points in a human brain, using dMRI and HARDI data. The basic idea is to define a composite energy functional, using a linear combinations of terms that relate to data, curve smoothness, and a prior shape model, and then use the gradient of this energy to iteratively optimize a contour. There are several novelties in this setup: (1) the data term is locally scale-invariant and measures only the agreement of the fiber direction with the given diffusion tensor field, (2) the length of the fiber is kept as a separate term, in order to have an additional control over fiber size, and (3) an external term involving statistical shape models, of fibers tracts connecting given regions, is used to improve optimization and interpretability. These shape models can come from training data developed using manual interventions or population atlases established from previous studies. The gradients of all the terms have analytical forms, making the gradient-based optimization very efficient. This framework is demonstrated successfully using simulated 2D tensor fields and 2D slices of volume dMRI data.

**Figure 12:**
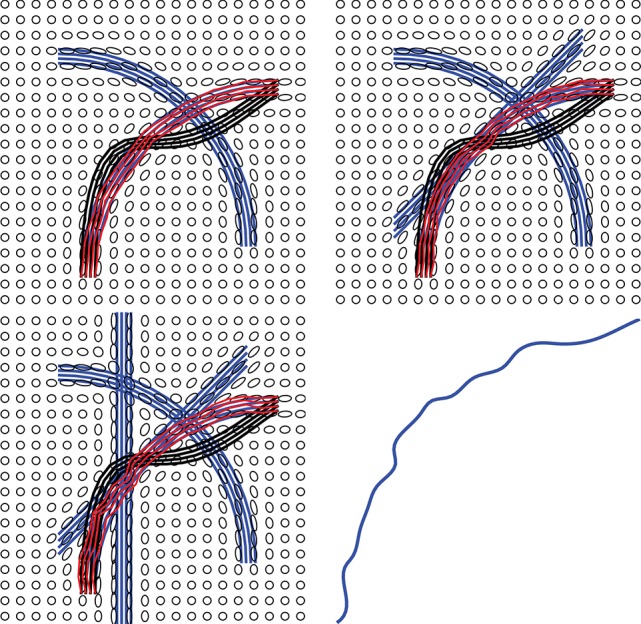
Examples showing that the proposed method can handle crossing and kissing fibers. Red lines are our tractograhy results, blue lines are ground truth and black lines are initializations. From upper left panel to bottom left panel, more and more crossing bundles are added into the simulation. The bottom right panel shows the shape prior used in our model.

One advantage of our method is that it can naturally handle crossing bundles since we construct the streamline as a whole object. Relying on the prior shape information, we can reconstruct a fiber curve that have similar geometry to the prior knowledge. Figure 12 illustrates one example that the proposed method is not sensitive to local fiber crossing. The blue lines are ground truth to generate the tensor field. From upper left to bottom left, more fibers were added to a region, which complicates the underlying tensor field. For the two selected regions, we initialize some black lines to connect them and the red lines are the final tractograhy results using our method. Those results indicates that our method can successfully reconstruct the fiber bundles in this challenge situation. The bottom right panel shows the shape prior that being used in our implementation.

However, the proposed Bayesian method needs to specify the starting and ending points for each extracted tract. To ensure that there is a tract between two ROIs, we currently rely on the atlas data. This procedure may end up with false positives, e.g., identifying a tract that does not exist. A future pruning procedure can be added as a post processing step, relying perhaps on the minimum energy as the reviewer has suggested. As another criterion, the diffusion profile along a tract can possibly be used as a feature to determine whether a tract is a false or a true positive.

As a future work, this framework can be naturally implemented using 3D dMRI data, and resulted tractography can be compared with some state of the art techniques.

## Author contributions

All authors listed, have made substantial, direct and intellectual contribution to the work, and approved it for publication. XD, ZZ, and AS have contributed in development of theory and computer implementation.

### Conflict of interest statement

The authors declare that the research was conducted in the absence of any commercial or financial relationships that could be construed as a potential conflict of interest.

## Appendix

### Lemma 1

In this section we derive an expression for the gradient of *E*_*data*_[β] with respect to β. Let *h* ∈ B be a perturbation to the curve β such that it is zero at the boundaries, i.e., *h* : [0, 1] → ℝ^2^ and *h*(0) = *h*(1) = 0. Since, $E_{data}\left[\beta +\epsilon h\right]=\int_{0}^{1}n_{\beta +\epsilon h}^{T}\left(t\right)M_{\beta \left(t\right)+\epsilon h\left(t\right)}^{-1}n_{\beta +\epsilon h}^{T}\left(t\right)dt$, the directional derivative of *E*_*data*_ in the direction of *h* is given by:

$$\begin{array}{l} \frac{d}{d\varepsilon }{|}_{\varepsilon =0}E_{data}[\beta +\varepsilon h]=\int_{0}^{1}(2n_{\beta }^{T}(t)M_{\beta (t)}^{-1}u_{\beta ,h}(t)\\ \;+n_{\beta }^{T}(t)\langle \langle \nabla_{x}M_{\beta (t)}^{-1},h(t)\rangle \rangle n_{\beta }(t))dt, \end{array}$$

where: $u_{\beta ,h}\left(t\right)=\frac{d}{d\epsilon }{|}_{\epsilon =0}\left(n_{\beta +\epsilon h}\left(t\right)\right)=\frac{1}{\left|\dot{\beta }\left(t\right)\right|}\left(I-n_{\beta }\left(t\right)n_{\beta }^{T}\left(t\right)\right)ḣ\left(t\right)\equiv A_{\beta }\left(t\right)ḣ\left(t\right)$. The last equality is used to define *A*_β_(*t*). We simplify the two terms one by one:

- **First Term**: Using integration by parts and using the boundary conditions *h*(0) = *h*(1) = 0, the first term becomes:
  $$\begin{array}{l} \int_{0}^{1}2n_{\beta }^{T}(t)M_{\beta (t)}^{-1}u_{\beta ,h}(t)dt=\int_{0}^{1}2n_{\beta }^{T}(t)M_{\beta }^{-1}(t)A_{\beta (t)}\dot{h}(t))dt\\ \;=-\int_{0}^{1}\langle 2\frac{d}{dt}\left(A_{\beta (t)}M_{\beta (t)}^{-1}n_{\beta }(t)\right)h(t)\rangle dt \end{array}$$
  Here
  $$\begin{array}{l} \frac{d}{dt}\left(A_{\beta (t)}M_{\beta (t)}^{-1}n_{\beta }(t)\right)=\frac{d}{dt}\left(\frac{1}{\left|\dot{\beta }(t)\right|}\left(I-n_{\beta }(t)n_{\beta }^{T}(t)\right)M_{\beta (t)}^{-1}n_{\beta }(t)\right)\\ \;=\frac{1}{\left|\dot{\beta }(t)\right|}\left(M_{\beta (t)}^{-1}{\dot{n}}_{\beta }(t)+\langle \langle \nabla_{x}M_{\beta (t)}^{-1},\dot{\beta }(t)\rangle \rangle n_{\beta }(t)\right)-\frac{{\dot{\beta }}^{T}(t)\ddot{\beta }(t)}{|\dot{\beta }(t){|}^{3}}M_{\beta (t)}^{-1}n_{\beta }(t)\\ \;-\frac{1}{\left|\dot{\beta }(t)\right|}\left({\dot{n}}_{\beta }(t)n_{\beta }^{T}(t)M_{\beta (t)}^{-1}n_{\beta }(t)+n_{\beta }(t)n_{\beta }^{T}(t)\langle \langle \nabla_{x}M_{\beta (t)}^{-1}\dot{\beta }(t)\rangle \rangle n_{\beta }(t)+2n_{\beta }(t)n_{\beta }^{T}(t)M_{\beta (t)}^{-1}{\dot{n}}_{\beta }(t)\right)\\ \;+\frac{{\dot{\beta }}^{T}(t)\ddot{\beta }(t)}{|\dot{\beta }(t){|}^{3}}n_{\beta }(t)n_{\beta }^{T}(t)M_{\beta (t)}^{-1}n_{\beta }(t),\text{where }{\dot{n}}_{\beta }(t)=\frac{d}{dt}n_{\beta }(t)=\frac{\ddot{\beta }(t)}{\left|\dot{\beta }(t)\right|}-\frac{\dot{\beta }(t){\dot{\beta }}^{T}(t)\ddot{\beta }(t)}{|\dot{\beta }(t){|}^{3}}. \end{array}$$
- **Second Term**: The second term can be rearranged as:
  $$\begin{array}{l} \int_{0}^{1}\langle \langle \langle tran(\nabla_{x}M_{\beta (t)}^{-1})n_{\beta }(t)n_{\beta }(t),h(t)\rangle \rangle \;dt \end{array}$$
  where $tran\left(\nabla_{x}M_{\beta \left(t\right)}^{-1}\right)$ is transpose of $\nabla_{x}M_{\beta \left(t\right)}^{-1}$.

Thus, the full gradient of *E*_*data*_ with respect to β is given by:

$$\begin{array}{l} -2\{\frac{1}{\left|\dot{\beta }(t)\right|}\left(M_{\beta (t)}^{-1}{\dot{n}}_{\beta }(t)+\langle \langle \nabla_{x}M_{\beta (t)}^{-1},\dot{\beta }(t)\rangle \rangle n_{\beta }(t)\right)-\frac{{\dot{\beta }}^{T}(t)\ddot{\beta }(t)}{|\dot{\beta }(t){|}^{3}}M_{\beta (t)}^{-1}n_{\beta }(t)\\ -\frac{1}{\left|\dot{\beta }(t)\right|}\left({\dot{n}}_{\beta }(t)n_{\beta }^{T}(t)M_{\beta (t)}^{-1}n_{\beta }(t)+n_{\beta }(t)n_{\beta }^{T}(t)\langle \langle \nabla_{x}M_{\beta (t)}^{-1}\dot{\beta }(t)\rangle \rangle n_{\beta }(t)+2n_{\beta }(t)n_{\beta }^{T}(t)M_{\beta (t)}^{-1}{\dot{n}}_{\beta }(t)\right)\\ +\frac{{\dot{\beta }}^{T}(t)\ddot{\beta }(t)}{|\dot{\beta }(t){|}^{3}}n_{\beta }(t)n_{\beta }^{T}(t)M_{\beta (t)}^{-1}n_{\beta }(t)\}+\langle \langle tran(\nabla_{x}M_{\beta (t)}^{-1}),n_{\beta }(t)\rangle \rangle n_{\beta }(t). \end{array}$$

### Lemma 2

Let's denote *f*_*p*_ as the ODF at *p* ∈ *D* and for simplicity, *f*(*t*) will be used to denote *f*_β(*t*)_(*n*_β_(*t*)) in the following derivation. In this section we derive an expression for the gradient of *E*_*data*_[β] with respect to β. Let *h* ∈ B be a perturbation to the curve β such that it is zero at the boundaries, i.e., *h* : [0, 1] → ℝ^2^ and *h*(0) = *h*(1) = 0. Since, $E_{data}\left[\beta +\epsilon h\right]=\int_{0}^{1}f\left(n_{\beta +\epsilon h}\left(t\right)\right)dt$, the directional derivative of *E*_*data*_ in the direction of *h* is given by:

$$\begin{array}{l} \frac{d}{d\varepsilon }{|}_{\varepsilon =0}E_{data}[\beta +\varepsilon h]=\int_{0}^{1}\nabla_{n_{\beta }}f(t)u_{\beta ,h}(t)dt, \end{array}$$

where: $u_{\beta ,h}\left(t\right)=\frac{d}{d\epsilon }{|}_{\epsilon =0}\left(n_{\beta +\epsilon h}\left(t\right)\right)=\frac{1}{\left|\dot{\beta }\left(t\right)\right|}\left(I-n_{\beta }\left(t\right)n_{\beta }^{T}\left(t\right)\right)ḣ\left(t\right)\equiv A_{\beta }\left(t\right)ḣ\left(t\right)$. Using integration by parts and using the boundary conditions *h*(0) = *h*(1) = 0, the term becomes:

$$\begin{array}{l} \int_{0}^{1}\nabla_{n_{\beta }}f(t)u_{\beta ,h}(t)dt=\int_{0}^{1}\nabla_{n_{\beta }}f(t)A_{\beta (t)}\dot{h}(t))dt=-\int_{0}^{1}\langle \frac{d}{dt}\left(A_{\beta }(t)\nabla_{n_{\beta }}^{T}f(t)\right)h(t)\rangle dt \end{array}$$

Here

$$\begin{array}{l} \frac{d}{dt}\left(A_{\beta (t)}\nabla_{n_{\beta }}^{T}f(t)\right)=\frac{d}{dt}\left(\frac{1}{\left|\dot{\beta }(t)\right|}\left(I-n_{\beta }(t)n_{\beta }^{T}(t)\right)\nabla_{n_{\beta }}^{T}f(t)\right)\\ \;=-\frac{{\dot{\beta }}^{T}(t)\ddot{\beta }(t)}{|\dot{\beta }(t){|}^{3}}\left(I-n_{\beta }(t)n_{\beta }^{T}(t)\right)\nabla_{n_{\beta }}^{T}f(t)-\frac{2}{\left|\dot{\beta }(t)\right|}{\dot{n}}_{\beta }(t)n_{\beta }^{T}(t)\nabla_{n_{\beta }}^{T}f(t)\\ \;+\frac{1}{\left|\dot{\beta }(t)\right|}\left(I-n_{\beta }(t)n_{\beta }^{T}(t)\right)\nabla_{n_{\beta }}^{2}f(t){\dot{n}}_{\beta }(t), \end{array}$$

where ${\dot{n}}_{\beta }(t)=\frac{d}{dt}n_{\beta }(t)=\frac{\ddot{\beta }(t)}{\left|\dot{\beta }(t)\right|}-\frac{\dot{\beta }(t){\dot{\beta }}^{T}(t)\ddot{\beta }(t)}{|\dot{\beta }(t){|}^{3}}.$.

Thus, the full gradient of *E*_*data*_ with respect to β is given by:

$$\begin{array}{l} -\frac{{\dot{\beta }}^{T}(t)\ddot{\beta }(t)}{|\dot{\beta }(t){|}^{3}}\left(I-n_{\beta }(t)n_{\beta }^{T}(t)\right)\nabla_{n_{\beta }}^{T}f(t)-\frac{2}{\left|\dot{\beta }(t)\right|}{\dot{n}}_{\beta }(t)n_{\beta }^{T}(t)\nabla_{n_{\beta }}^{T}f(t)+\frac{1}{\left|\dot{\beta }(t)\right|}\left(I-n_{\beta }(t)n_{\beta }^{T}(t)\right)\nabla_{n_{\beta }}^{2}f(t){\dot{n}}_{\beta }(t). \end{array}$$
